# Current treatment of systemic lupus erythematosus: a clinician's perspective

**DOI:** 10.1007/s00296-023-05306-5

**Published:** 2023-05-12

**Authors:** Pawlak-Buś Katarzyna, Schmidt Wiktor, Dudziec Ewa, Leszczyński Piotr

**Affiliations:** 1 Department of Internal Medicine, Poznań University of Medical Sciences, Poznań, Poland; 2 Department of Rheumatology, Systemic Connective Tissue Diseases and Immunotherapy of Rheumatic Diseases, J. Struś Municipal Hospital, Poznań, Poland; 3 Doctoral School, Poznań University of Medical Sciences, Poznań, Poland

**Keywords:** Systemic lupus erythematosus, Antimalarials, Glucocorticoids, Immunosuppressants, Biological therapy, JAK inhibitors

## Abstract

Systemic lupus erythematosus (SLE) is a chronic autoimmune disease. Its variable course makes it difficult to standardize patient treatment. This article aims at a literature review on available drugs for treating SLE and on drugs that have shown therapeutic effects in this disease. The PubMed/MEDLINE electronic search engine was used to identify relevant studies. This review presents the current therapeutic options, new biological therapies, and combination therapies of biologics with standard immunosuppressive and immunomodulating drugs. We have also underlined the importance to implement the treat-to-target strategy aimed at reducing or discontinuing therapy with glucocorticosteroids (GCs). The awareness of the benefits and risks of using GCs helps in refining their dosage and thereby obtaining a better safety profile. The advent of biological targeted therapies, and more recently, low-molecular-weight compounds such as kinase inhibitors, initiated numerous clinical trials in SLE patients and led to the approval of two biological drugs, belimumab, and anifrolumab, for SLE treatment. Progress in the treatment of SLE was reflected in the 2019 and 2021 recommendations of the European Alliance of Associations for Rheumatology (EULAR). However, a mass of recent clinical research data requires continuous consolidation to optimize patient outcomes.

## Introduction

Systemic lupus erythematosus (SLE) is a chronic autoimmune disease with a multidimensional clinical picture. Due to the unpredictable course of this disease and the involvement of many organs, treating SLE remains challenging for physicians. Decades of experience in treating patients with SLE have improved our understanding of the mechanisms of action and therapeutic effects of antimalarial drugs and glucocorticosteroids (GCs). Due to the spectacular and rapid action of GCs in dampening disease exacerbations (flares), they have been used for years as basic drugs in SLE treatment. However, it is known that these drugs cause serious complications. More recent studies indicate that the administration of higher GC doses is, in many cases, unnecessary and associated with organ damage [1]. A wider range of available treatment options and more effective treatment of disease comorbidities, especially cardiovascular disease and infections, improved patient outcomes and reduced mortality [2, 3]. Other common serious complications of SLE requiring appropriate management are atherosclerosis, end-stage renal disease (ESRD), dyslipoproteinemia, diabetes mellitus, osteoporosis, cataracts, and chronic fatigue.

The current treatment strategy for SLE is based on the treat-to-target principle and focuses on achieving a defined state of remission or low disease activity. This treatment approach may result in increased clinical benefits for patients with SLE [4–7]. It should be realized by (i) induction of remission, (ii) consolidation of remission, and (iii) remission maintenance. The treat-to-target strategy involves the use of immunosuppressive treatment and biologics to achieve the goal of low disease activity or, preferably, remission without the need for GCs. In this context, the objective assessment of disease activity is essential (according to the most recommended tools, such as the SLE Disease Activity Index [SLEDAI]) to establish a long-term treatment strategy (taking into account risk factors such as cardiovascular risk [CVR]) and aiming at reducing or even stopping treatment with GCs. Generally, the treatment of patients with SLE should follow the recommendations of the European Alliance of Associations for Rheumatology (EULAR) [8, 9]. However, the final choice of treatment is a shared decision of the patient and the physician based on a weighted risk–benefit assessment for an individual patient. This choice is based on the physician’s experience and considers the disease activity, the risk of flare, and damage accrual.

The aim of this article was to perform a literature review on drugs for treating SLE that are available or that have shown therapeutic effects. The PubMed/MEDLINE electronic search engine was used to identify relevant studies. The search was focused on original studies, recommendations, reviews, and systematic reviews presenting state-of-the-art knowledge in the field and on the most up-to-date studies demonstrating novel therapeutic possibilities. Therefore, this review presents the current therapeutic options, especially in terms of new biological therapies and combination therapies of biologics with standard immunosuppressive and immunomodulating drugs. We have underlined the need to implement the treat-to-target strategy aimed at reducing and discontinuing GC therapy which is often abused and generates organ complications.

## Medicines used to treat SLE

SLE is characterized by the presence of multiple autoantibodies against nuclear components and systemic inflammation, which lead to the damage of multiple organs. Abnormal maturation and activation of B-cells play a pivotal role in the immunopathogenesis of SLE in both antibody-dependent and antibody-independent manners [10]. Anti-inflammatory and immunosuppressive drugs are used to treat immunological disturbances in SLE. These include non-specific anti-inflammatory and immunosuppressive drugs, such as antimalarial drugs, GCs, non-corticosteroid immunosuppressants, and targeted therapies. The targeted therapies directly or indirectly affect B-cell survival and activation, leading to the depletion of B-cells or inhibition of their activity. The characteristics of non-corticosteroid immunosuppressants and biologics used in SLE treatment are presented in Table 1.

**Table 1:** Non-corticosteroid immunosuppressants and biologics characteristics in SLE

	Immunosuppressive agent	Dose	Main indications in SLE	Adverse events	Special issue
Non-steroidal immunosuppressants	CYC	Low dose 500 mg, iv, biweekly, 4 times High dose 0.75–1.0 mg/m^2^, iv, monthly, 6 times	Severe organ involvement: LN NPSLE Vasculitis	Cystitis (especially high doses) Gastrointestinal Hematological	Teratogenic effect Premature ovarian failure Malignancies To prevent bladder toxicity—Mesna administration For overall less toxicity—regimen according to Euro-Lupus [51]
	MMF	2.0–3.0 g/day, orally	Hematologic Skin LN in induction and maintenance therapy	Gastrointestinal Hematological	Teratogenic effect
	Calcineurin inhibitors				
	CsA	3.0—5.0 mg/kg/day, orally	LN Skin Hematologic	Nephrotoxicity Metabolic (hypertension, hyperglycemia, hyperlipidemia) Hirsutism	Safe during pregnancy and lactation Continuing with folic acid during pregnancy
	Voclosporin	23.7 mg, twice daily, orally	LN	Hypertension Nephrotoxicity Serious infections Malignancies	Combination therapy with MMF in LN Dose adjustment based on eGFR Advantages compared with CsA and tacrolimus with respect to dosing and tolerability
	Tacrolimus	0.2–0.3 mg/kg/day, orally	LN	Nephrotoxicity Cardiomyopathy	Combination therapy with MMF in LN with nephrotic proteinuria
	AZA	1.0–5.0 mg/kg/day, orally Pregnancy and lactation: ≤ 2.0 mg/kg/day, orally	Hematologic Skin LN in maintenance therapy	Gastrointestinal Hepatotoxicity Hematological	Drug interactions with allopurinol Safe during pregnancy and lactation Mild GCs-sparring effect
	MTX	7.5–25 mg/week, orally/sc	Joints Skin Serositis	Gastrointestinal Hepatotoxicity Hematological	Teratogenic effect Mild GCs-sparring effect Use with caution in elderly patients and in patients with reduced GFR (< 30 ml/min)
Biologics	Belimumab	Iv: 10.0 mg/kg on days 0, 14 and 28, then every 28 days sc: 200 mg/week In LN: 400 mg/week, 4 times; then 200 mg/week	Skin Joints LN	Infections Depression Progressive multifocal encephalopathy	Complementary treatment in seropositive moderate to severe SLE Not recommended in severe NPSLE FDA-approved to treat seropositive, moderate SLE in children 5–17 years of age
	Anifrolumab	300 mg, iv, every 4 weeks	Skin Joints	Infections, especially herpes zoster	Complementary treatment in seropositive moderate-to-severe SLE GCs-sparing effect
	Rituximab	500–1000 mg, iv, on days 0 and 14, the next course to be administered after 6 months	Refractory LN NPSLE	Progressive multifocal encephalopathy Infections	Drug off label Different administration schedules

AZA azathioprine, CsA cyclosporine, CYC cyclophosphamide, eGFR estimated glomerular filtration rate, GCs glucocorticosteroids, FDA Food and Drug Administration, GFR glomerular filtration rate, iv intravenous, LN lupus nephritis, MMF mycophenolate mofetil, NPSLE neuropsychiatric systemic lupus erythematosus, sc subcutaneous

### Antimalarials

The treatment of autoimmune diseases with antimalarials has a long history, and chloroquine (CQ) and hydroxychloroquine (HCQ) are still used to treat patients with SLE. The main effects of antimalarial drugs are the inhibition of lysosomal activity and autophagy, inhibition of pro-inflammatory cytokine signaling and secretion, inhibition of T-cell proliferation, and blocking of Toll-like receptors [11–13].

The activity of SLE and the accrual of organ damage can be significantly reduced with chronic HCQ treatment, provided that the patient's blood HCQ concentration remains at 800–1000 ng/ml [14, 15]. HCQ also delays the appearance of disease flares [16, 17]. However, the expected clinical results are difficult to achieve due to poor patient compliance; 7–29% of patients treated with this drug had HCQ < 200 ng/ml [18]. This observation demonstrates the need to monitor patients' adherence to HCQ therapy. In lupus nephritis (LN), a severe phenotype of lupus, antimalarials reduce the risk of renal flare, ESRD, and death [19, 20]. Other benefits of using antimalarial drugs include prolonged survival and reduction of damage accrual [21–23]. In the context of organ protection, it is important that antimalarials use allows reduction of GC dose [24]. HCQ has also been shown to have a beneficial effect on lipid profile and glycemic control. It can improve endothelial function and inhibit platelet aggregation and arachidonic acid release. The other benefit of antimalarial therapy is diminishing the risk of developing cardiovascular disease by reducing the number of thrombotic events. HCQ has also protective effects against severe bacterial and viral infections and their complications [25, 26]. HCQ treatment is recommended as the background therapy for all patients with SLE without contraindications to this drug [8]. These contraindications are limited to retinopathy and cardiomyopathy and relate to high doses of HCQ. Therefore, in SLE, a dose of HCQ > 5 mg/kg body mass (corresponding to an HCQ cumulative dose of > 1000 g) is not recommended [27]. Maculopathy develops in 2% and 0.1% of patients with SLE within 10 years of CQ or HCQ treatment, respectively. To detect asymptomatic, early, and reversible retinopathy, careful monitoring of patients undergoing this therapy is necessary [28]. HCQ can be administered to reduce the rates of lupus flares in pregnant women [29]; however, the scarcity of data on pharmacokinetics and posology of this drug in pregnant women does not allow for indication of recommended HCQ dosage [30].

Quinacrine, used to treat skin lesions in SLE, does not increase retinopathy risk. This drug inhibits the production of TNF-α and INF-α by dendritic cells and monocytes [31]. A synergistic effect of HCQ and quinacrine on the improvement of cutaneous lupus erythematosus has been reported [32].

### Glucocorticoids

Glucocorticoids can rapidly control SLE activity and remain the cornerstone of SLE therapy. The potency of GCs in dampening inflammation is associated with a broad spectrum of effects on the immune system; GCs reduce the expression of cytokines and adhesion molecules, inhibit leucocyte traffic, and their access to inflammation sites, and interfere with leucocyte, fibroblast, and endothelial cell functions [33]. Observational studies indicate that up to 88% of patients with SLE are treated with GCs, and more than half continue this therapy for a long time [34, 35]. A significant proportion of the early and late damage during disease treatment could be attributed to GC therapy [35, 36]. These findings are of great importance because irreversible organ damage has been reported to be a predictor of morbidity and mortality in SLE [37]. Therefore, using GCs more restrictively should help prevent serious complications in patients with SLE. According to the current recommendations, the use of CGs should be limited or completely discontinued depending on the activity of the disease, the duration of treatment, and the desired effects of GC treatment [5].

After diffusion into the cell, GCs can activate transcription factors either directly (via the genomic pathway) or indirectly (via the non-genomic pathway) [38]. In the genomic pathway, GC binds to the cytosolic receptor (GR), thereby becoming activated. The GC-GR complex then translocates to the cell nucleus, where it induces the transcription of genes encoding anti-inflammatory mediators. The effect of the genomic pathway is evident hours or days after its activation. However, the clinical effects of GC may be faster as high systemic doses of methylprednisolone can quickly alleviate the symptoms of a flare. The rapid effects of GCs are related to the activation of the non-genomic pathway. In this mechanism, GC binding allows the GR to interact with intracellular proteins as well, leading to rapid inhibition of inflammatory mediators such as arachidonic acid. The recruitment potential of a given pathway depends on the type and dose of GC. Compared to other steroids, methylprednisolone or dexamethasone are 10–15 and 20 times more potent in inducing a non-genomic pathway, respectively [39]. When using a dose above 30–100 mg/day of prednisone equivalent, the saturation of the cytosolic GR is ~ 100%, and the genomic pathway is fully operational. In contrast, the non-genomic pathway operates at clinically relevant levels at doses of > 100 mg/day of prednisone equivalent [40]. The usual dose of 1 mg/kg/day for treating SLE exacerbations is empirical and is currently not recommended since it was observed that the resulting response rates after using high initial prednisone doses of 1 mg/kg/day did not differ significantly from those obtained for pulse methylprednisolone in lower doses (0.3 and 0.5 mg/kg day), whether CYC or MMF was used [40]. This is an important observation as it has recently been shown that many side effects are related to the dose of GC used and the activation of the genomic pathway. Serious toxicity begins at 7.5 mg/day of prednisone equivalent, which is the borderline between low and medium pharmacological doses. Data from observational and clinical studies have shown that low-dose methylprednisolone pulses (125–500 mg daily) are potent treatments for severe acute lupus exacerbations with few associated side effects [41, 42]. However, even with low GC doses, the risk of cataracts, ischemic heart disease, osteoporosis, and fractures in long-term GC treatment may increase [43]. Therefore, lower GC doses (i.e., < 5 mg/day of prednisone equivalent) should be considered when it is not possible to discontinue long-term GC therapy. Concomitant use of antimalarial and immunosuppressive drugs may help to maintain a low GC dose or even to withdraw it [44, 45].

It should be noted that the definition of remission also considers the dose of GC the patient is taking; the use of GC in a daily dose of above 5 mg excludes the occurrence of remission of SLE in a patient [5]. According to recommendations of the European Renal Association-European Dialysis and Transplant Association for LN, withdrawal of GCs is suggested in patients being in remission for 3 years [46]. However, in real life, clinicians prefer to withdraw GC after 5 years of sustained remission while maintaining immunosuppressive therapy, particularly when the disease has a relapsing–remitting course with severe organ involvement [34]. The greatest concern for clinicians prior to discontinuing GC therapy in patients with inactive SLE is the risk of disease exacerbation. A recent meta-analysis of 738 lupus patients after discontinuation of GC revealed an increased risk of clinical flare (although not a major flare) and less organ damage assessed by the SLICC/ACR damage index [47]. Other recently published data indicate that gradual withdrawal of GC is safe in clinically inactive SLE and is associated with fewer disease flares and less damage accrual [48]. In the case of prolonged exposure of patients to GCs, an increase in the frequency of infections should be taken into account [26, 49].

### Non-corticosteroid immunosuppressants

Non-corticosteroid immunosuppressants target different B-cell populations. Cyclophosphamide (CYC) preferentially depletes less mature B-cells (naïve and pre-switching memory B-cells), while mycophenolate mofetil (MMF) depletes circulating plasmablasts. Azathioprine (AZA) is less potent in suppressing naïve and memory B-cells than MMF [50]. Non-corticosteroid immunosuppressants constitute the basic therapy for reducing SLE activity. They are used to initiate and maintain therapy. The type and dose of immunosuppressants should be adapted to the activity of lupus, the type, and the severity of the organ involved or manifestations. The most commonly used non-corticosteroid immunosuppressants in SLE are AZA, calcineurin inhibitors, methotrexate (MTX), and MMF. Life-threatening involvement of organs and systems, such as in LN and neuropsychiatric lupus, requires more aggressive parenteral treatment with CYC or MMF in combination with GCs. The teratogenic properties of CYC, MMF, and MTX should be considered when treating young women with maternity plans [29].

CYC interferes with DNA and blocks replication in proliferating cells, including B-cells. High doses of intravenous CYC in combination with GC are used as first-line therapy to induce remission in proliferative LN. Data from the Euro-Lupus Nephritis Trial show that remission-inducing treatment with a low dose of CYC gives clinical results comparable to those obtained with high-dose treatment but has a better safety profile [51]. These observations may influence the management of other difficult-to-treat cases of SLE, such as patients with vasculitis, and may reduce mortality. However, other randomized clinical trials of proliferative LN have shown little to no difference in complete remission rate in patients treated with intravenous CYC or MMF as induction therapy [52].

MMF, an orally administered prodrug, is metabolized in the liver to the active mycophenolic acid molecule. Mycophenolic acid is a potent, reversible inhibitor of inosine-5′-monophosphate dehydrogenase, an enzyme essential for the de novo synthesis of guanosine-5′-monophosphate. The use of MMF to inhibit DNA replication affects especially T cells and B cells, as they rely almost exclusively on de novo purine synthesis. MMF is recommended for the treatment of patients with LN. This drug is effective in inducing remission and as a long-term maintenance therapy. Compared to CYC, the use of MMF may lead to complete renal remission more frequently with a more favorable side effect profile [52]. In maintenance therapy, MMF was shown to be superior to AZA in preventing relapse in LN [52]. However, recent data suggest that the risk of LN relapse may be related to changes in B cell subpopulations and different B cell signatures in patients [50].

Calcineurin inhibitors affect B cells indirectly by inhibiting the activity of T cells. These drugs bind to the cytosolic protein cyclophilin and inhibit calcineurin, which prevents the activation of the NF-AT transcription factor. The traditional drug from this group is cyclosporin A (CSA), which has a beneficial effect and a good safety profile in pregnant women with SLE. Voclosporin (already registered in the US and EU) is a novel calcineurin inhibitor that, when combined with MMF and GC, allows a better complete renal response rate than MMF and GC alone in patients with active LN. The advantage of using voclosporin is fewer side effects, such as hypertension, increased mortality, and worsening renal function [53, 54]. Tacrolimus is another calcineurin inhibitor that is used in severe LN. Long-term data of a randomized controlled trial confirmed non-inferiority of tacrolimus to MMF as induction therapy of LN assessed by response rate to treatment and rate of flares [55].

AZA is mainly used as maintenance therapy for moderate-to-severe lupus. AZA is a prodrug that converts into 6-mercaptopurine and interferes with DNA replication and purine synthesis in lymphocytes. Animal studies have shown that the immunosuppressive effects of AZA are dose-dependent. Higher doses of AZA are needed for the effective suppression of humoral immunity than for the suppression of cellular immunity [56, 57]. According to a systemic analysis of nine studies, maintenance treatment of proliferative LN with AZA may increase the relapse rate compared to MMF (MMF risk ratio 1.75, 95% CI 1.20 to AZA 2.55) [52]. It was shown that the depletion of B-cells at an earlier stage of their development results in better treatment outcomes which may explain the stronger immunosuppressive effect of MMF than AZA [50]. AZA can be continued during pregnancy [29].

MTX may be effective when the musculoskeletal and mucocutaneous domains are involved in mild-to-severe SLE [9]. Results of the study involving 41 patients with cutaneous disease indicate that MTX may be superior to placebo in terms of complete clinical response (absence of malar/discoid rash) at 6 months of follow-up [58].

### Biologics

**Fig. 1:**
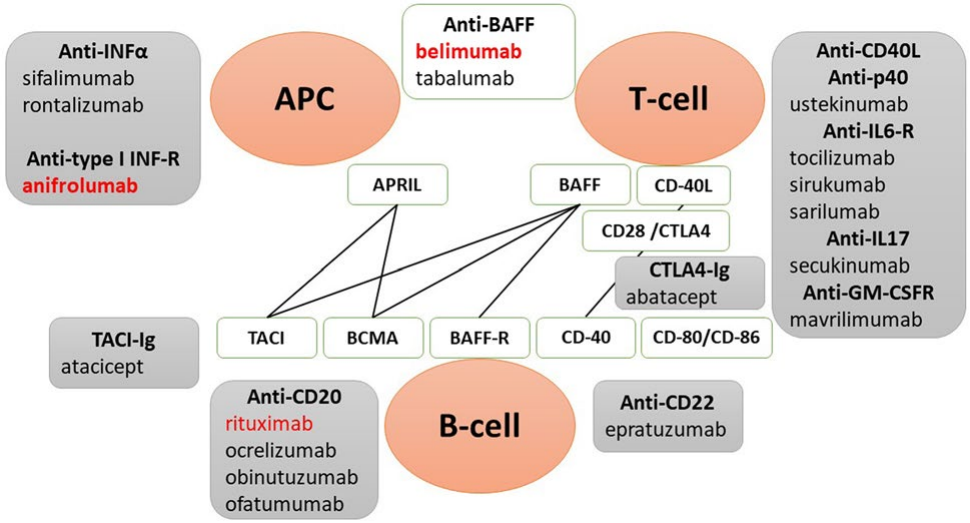
Treatment strategies affecting B cells in SLE. The diagram shows the molecules (in white fields) involved in B cell stimulation and interaction with other cell types (dendritic cells, T cells). These molecules are therapeutic targets in SLE; blocking their activity may help to normalize B cell activity in SLE. The shaded boxes show the biological drugs currently available that affect the activity of B cells, T cells, and APCs. Biological drugs marked in red are approved for treating patients with SLE or are used off-label (RTX). APC antigen-presenting cell, BAFF (BlyS) B lymphocytes stimulator, APRIL ligand-inducing B cell proliferation, TACI transmembrane activator-1 and calcium modulator and cyclophilin ligand-interactor, BCMA B cell maturation antigen, RTX rituximab

The biologics approved for treating patients with SLE are belimumab and anifrolumab. Rituximab (RTX) is used off-label, and many other biological drugs are evaluated in clinical trials. Biologics should be considered in persistently active or recurrent SLE (Fig. 1).

#### Anti-BlyS agents

**Fig. 2:**
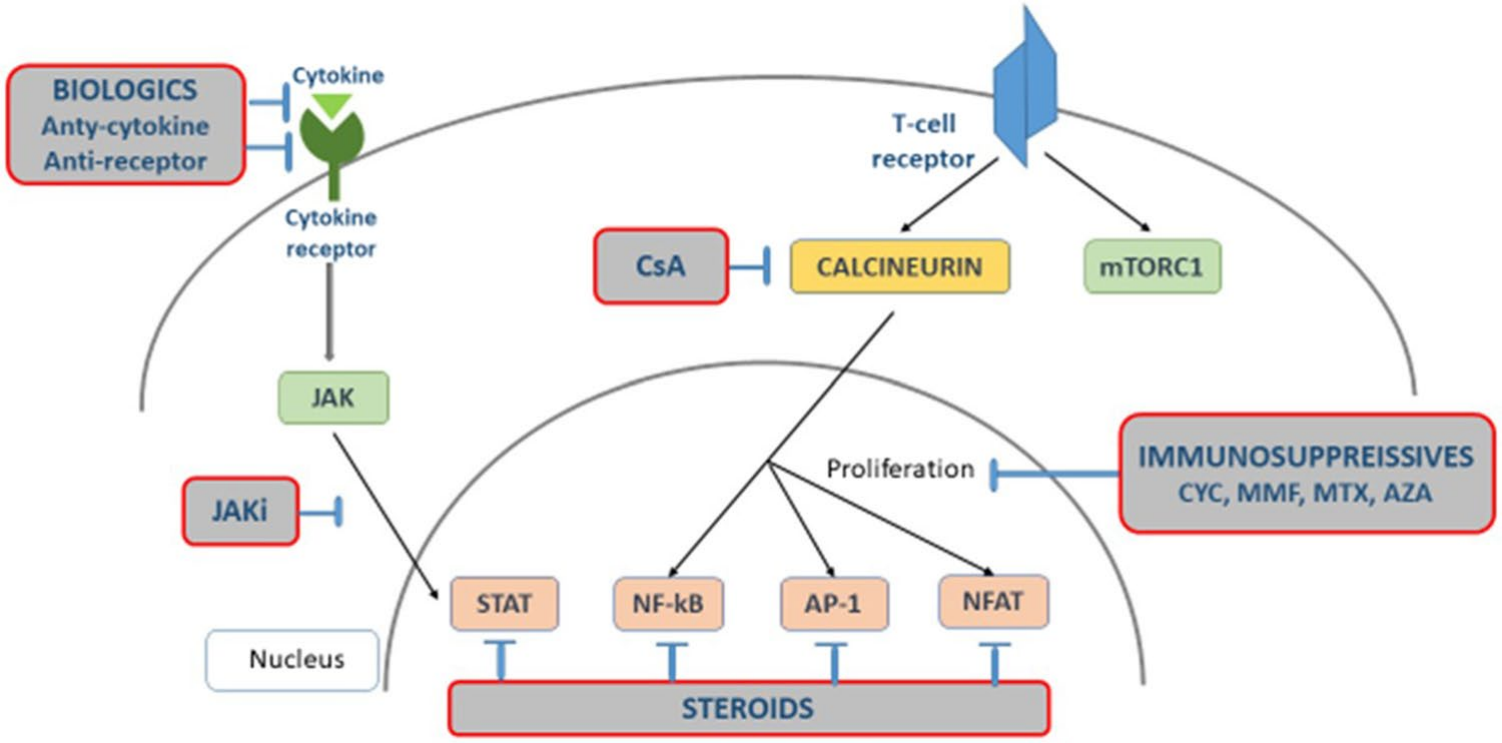
Therapeutic targets in SLE. The diagram shows the cell signaling pathways that drugs interfere with. CsA cyclosporine A, mTORC1 mammalian target of rapamycin complex, JAK Janus kinase, JAKi JAK inhibitor, STAT signal transducer and activator of transcription, NF-kB nuclear factor κB, AP-1 activator protein 1, NFAT nuclear factor of activated T cells, CYC cyclophosphamide, MMF mycophenolate mofetil, MTX methotrexate, AZA azathioprine

Two members of the TNF superfamily, B cell stimulator (BlyS) and ligand-inducing B cell proliferation (APRIL), can support autoreactive B cell survival and autoantibodies production in SLE [59]. BlyS exists in a soluble and membrane-bound form and is produced by dendritic cells and macrophages. Elevated serum levels of BlyS were found in patients with SLE. Moreover, both INF-α and INF-γ, deregulated in SLE, stimulate BlyS expression. BlyS can bind to three receptors: BR3 (BAFF-R), transmembrane activator-1 and calcium modulator and cyclophilin-interaction (TACI), and B-cell maturation antigen (BCMA) (Fig. 2). Expression of BlyS receptor types vary across different subsets of B cells. Mature B cells express BR3 and TACI, while plasma cells express BCMA. Thus, the biological effects elicited by BlyS depend on the type of receptor to which it binds. Data from animal models suggest that increased soluble BlyS levels in SLE may stimulate the production of anti-double-stranded DNA (dsDNA) antibodies. Moreover, autoreactive antibodies can be produced independently of T-cells when an excessive amount of BlyS is present [59]. APRIL is expressed by bone marrow stromal cells and supports the survival of plasma cells [59].

Belimumab is a human anti-BlyS monoclonal antibody (administered intravenously or subcutaneously) that was approved in 2011 in the US and Europe for the treatment of adult SLE and in 2019 for the intravenous treatment of pediatric lupus patients (ages 5–17). Belimumab binds to soluble BlyS. Two international, multicenter, randomized, placebo-controlled phase III trials, BLISS-52 and BLISS-76, demonstrated the efficacy of belimumab in serologically active moderate-to-severe lupus [60, 61]. BLISS-LN compared intravenous belimumab versus placebo in patients with active LN Class III, IV, and V on background therapy (GC and MMF or CYC). Significantly more patients had a complete renal response, including a reduction in urine protein to creatinine ratio to < 0.5 and no treatment failure at week 104 in the belimumab group than in the placebo group (odds ratio 1.7; 95% CI 1.1–2.7). The renal response was also higher and occurred earlier in patients treated with belimumab [62]. Subcutaneous administration of belimumab was studied in the BLISS-SC study. Patients with LN improved in renal parameters and allowed for tapering GCs doses [63]. Clinical observations indicate a slow disease response rate and a long onset of belimumab efficacy. This phenomenon is related to the slow turnover rate of BlyS and the slow off-time for BlyS and its receptor, which limits the drug’s action. It was also found that belimumab reduces mortality in patients with SLE (0.4/100 person-years) compared to the general population (1.63/100 person-years) [64]. In addition, the adverse event rate with belimumab treatment was acceptable. Therefore, belimumab is considered an effective and safe biological therapy and is expected to be used both for remission induction in refractory SLE and maintenance therapy to reduce the dose of GC.

Another fully human monoclonal antibody against BlyS is tabalumab. Tabalumab binds to both soluble and membrane-bound BlyS. The results of the two phase-III studies, ILLUMINATE-1 and -2, showed higher rates of disease responses (according to SRI-5) in the serologically active subgroup of patients, and a significant decrease in anti-dsDNA antibodies, increases in C3 and C4 levels, and reductions in total B-cells and immunoglobulins after treatment with tabalumab. However, the clinical response to tabalumab was insufficient [65, 66].

The premise for the development of atacicept was the assumption that blocking BlyS and APRIL simultaneously in SLE might be more effective than neutralizing BlyS alone. Atacicept is a soluble human recombinant fusion protein consisting of IgG1-Fc and the extracellular domain of TACI and is administered subcutaneously. Atacicept, through the extracellular domain of TACI, binds and neutralizes BlyS and APRIL [67]. It demonstrated encouraging effects in serologically active patients with SLE (phase IIb ADRESS II study and post hoc analysis), but due to severe infections during phase III, the APRIL-SLE was suspended [68, 69].

#### B cells depletion antibodies

B cell loss can be induced using monoclonal antibodies targeting their surface molecules CD20, CD22, and CD19. While no such drug is currently approved for treating SLE, numerous studies are ongoing.

Anti-CD20 monoclonal antibodies are effective in the treatment of hematological cancers and RA. The profound reduction in the CD20-expressing B cell populations results from antibody-dependent cellular cytotoxicity (ADCC), complement-dependent cytotoxicity, and apoptosis [70]. One such drug is rituximab (RTX), a chimeric monoclonal antibody approved for treating rheumatoid arthritis and granulomatosis with polyangiitis. Given the crucial role of B-cells in the pathogenesis of SLE, initial results from clinical trials have been disappointing. In the EXPLORER trial of patients with moderate-to-severe SLE, there were no differences between placebo and rituximab in achieving the primary and secondary endpoints [71]. However, in that study, a sensitive cutoff was used for non-response. In addition, aggressive background treatment might have masked the RTX effects. In patients with LN treated with RTX, response to treatment was greater in patients with greater anti-dsDNA and C3/C4 levels reduction. However, this medication did not improve clinical outcomes after 1 year of treatment [72]. Interestingly, considerable variability in peripheral blood B cell depletion among patients with LN was reported, and the achievement of complete peripheral depletion, its rapidity, and duration were associated with the improvement of renal function [73]. Variability in response among patients with SLE may be related to the formation of human anti-chimeric antibodies, which are correlated with poor B cell depletion [74]. Although the use of RTX in treating patients with SLE is not approved, it is used off-label in difficult-to-treat cases, especially in severe LN and lupus with neuropsychiatric symptoms [8]. A review of uncontrolled studies and cases involving 188 SLE patients who received RTX as an off-label revealed that 91% of patients improved in at least one lupus domain [75]. Clinical improvement was also noted in patients with neuropsychiatric lupus who were treated with RTX. However, 45–60% of these patients experienced a relapse within 17 months of RTX therapy [76, 77].

The next-generation humanized anti-CD20 monoclonal antibodies are ocrelizumab, obinutuzumab, and ofatumumab. They emerged to overcome the immunogenic activity of the chimeric antibody [78]. Although the novel anti-CD20 monoclonal antibodies can induce anti-human antibodies, they bind to the Fc receptor on B-cells with higher affinity and thus exert a more potent cytotoxic effect (CDC and/or ADCC).

Monoclonal antibodies targeting other B cell surface molecules are also tested in SLE, but no significant therapeutic effects have been yet obtained. Obexelimab (anti-CD19 antibody) did not show very encouraging effects in phase II in moderate SLE [79]. The humanized antibody epratuzumab binds to CD22 and transduces the negative signal leading to the inhibition of B-cell activity [80]. However, treating patients with moderate or severely active SLE with epratuzumab did not improve response rates over that observed in the placebo [81]. The high response rates to placebo after optimizing standard treatment and a suboptimal dosing regimen of epratuzumab may have resulted in trial failure.

#### Anti-INF I receptor antibodies

Interferon signature is a common SLE feature [82]. It is associated with increased levels of INFα in active disease, especially LN and neuropsychiatric lupus. Production of INFα in SLE can be induced by Toll-like receptors 9 (TLR9) or TLR7 upon binding their respective ligands. Ligands for TLR9 and TLR7 may be free DNA or RNA, respectively, present in SLE due to the ineffective removal of apoptotic bodies or the formation of extracellular neutrophil traps [82]. Given the vital role of type I INF in the pathogenesis of SLE, the results of numerous clinical trials with anti-INFα antibodies have been disappointing. Anifrolumab has recently been approved in the US for treating adult patients with moderate to severe SLE who receive standard treatment. Anifrolumab is a human monoclonal antibody against the type I INF receptor. This drug blocks signals induced by the type I IFNs. Anifrolumab was examined in placebo-controlled studies in patients with moderate-to-severe SLE undergoing standard therapy with GCs, immunosuppressants, and antimalarials. In the phase II MUSE study, anifrolumab significantly reduced disease activity (assessed using SRI-4, modified SRI, BICLA, and BILAG 2004) compared to placebo, with greater effect size in patients with a high IFN signature [83]. In phase III of the randomized, placebo-controlled TULIP clinical trial, patients with moderate to severe SLE, excluding LN, were involved. TULIP-2 study demonstrated the efficacy of anifrolumab, which was reflected in an increased BICLA (BILAG-based Combined Lupus Assessment) disease response rate, and a reduction in skin disease severity. The GC-sparing effect was also achieved with anifrolumab treatment [84].

#### Anti-IL-12/IL23 p40 antibody

T-cell abnormalities in SLE manifest as abnormal cytokine production. The cytokines IL-12 and IL-23 promote inflammation by stimulating Th1 cells and producing IL-17 [85, 86]. Ustekinumab was designed to suppress the activity of IL-12 and IL-23 by binding to their shared p-40 receptor subunit, thereby blocking the interaction of these cytokines with their respective receptor. Ustekinumab is approved for treating psoriasis (PS) and psoriatic arthritis (PsA). Phase II clinical trial of ustekinumab in active SLE showed a satisfactory response rate expressed by SLEDAI-2 K Responder Index-4, which was maintained for up to one year. Phase III of this clinical trial is ongoing [87].

### Combination therapy

Resistance to conventional treatment with GCs and non-corticoid immunosuppressants is common among patients with SLE [88]. Biologics have become an alternative and are being assessed in clinical trials in patients whose disease is not sufficiently controlled by conventional drugs. Sequential therapy can help improve the effectiveness of B cell depletion. Treatment with RTX and CYC, followed by belimumab, was evaluated in the CALIBRATE study in refractory or recurrent LN. This treatment strategy prevented autoreactive B-cell re-emergence but did not lead to sufficient clinical improvement [89]. However, in another phase II clinical trial, treatment with belimumab followed by RTX significantly reduced levels of serum anti-dsDNA antibodies and the risk of severe flare in patients with SLE who were refractory to conventional therapy [90]. A randomized, controlled phase III trial of SynBioSe-2 is ongoing to investigate the long-term clinical and immunological efficacy of the combination of belimumab followed by RTX in LN. Interestingly, belimumab was also used as maintenance therapy in two SLE cases with refractory renal and pulmonary manifestations rescued by bortezomib as induction of remission [91].

### Janus kinases inhibitors

The emergence of low-molecular-weight compounds in the treatment armamentarium allowed researchers to interfere directly with the intracellular cytokine signaling pathways.

Interaction of the proinflammatory cytokine with its membrane receptor triggers intracellular signaling that leads to gene transcription and inflammatory mediators’ production. Intracellular signal transmission depends on the activity of enzymes—kinases that phosphorylate signaling molecules [92]. In treating rheumatic diseases, 4 members of the Janus kinases family (JAK1, JAK2, JAK3, and tyrosine kinase-2; TYK2) and their 7 downstream signal transducers and activators of transcription (STATs) are currently of greatest interest. JAK inhibitors (JAKi) bind to the enzymatically active domain of JAK, thereby blocking its activity and cytokine-induced signal transduction. Baricitinib is an oral JAK1 and JAK2 inhibitor approved for the treatment of RA. In the phase IIb randomized multicenter placebo-controlled trial, baricitinib significantly improved the arthritis symptoms and resolved skin rash in patients with active SLE with skin or joint manifestations. Phase III of the clinical trial of baricitinib in SLE is ongoing [93]. TYK2 mediates the functional responses to IL-23, IL-12, and type I IFN stimulation. Selective TYK2 inhibitors might benefit patients with PS, PsA, inflammatory bowel disease (IBD), and SLE [94]. The TYK2 inhibitor, deucravacitinib, was effective in reducing SLE activity and tender and swollen joint counts in patients in an international, randomized, double-blind, placebo-controlled phase II clinical trial [95] and warrants further phase III research trials.

### Bruton’s tyrosine kinase inhibition

Bruton's tyrosine kinase (BTK) is a cytoplasmic signaling molecule involved in the development, survival, and activation of B cells. Fenobrutinib is an orally highly selective BTK inhibitor that reversibly binds its target. It was effective in reducing CD19-positive B cells and antibody production but was not sufficiently effective in randomized, placebo-controlled phase II trials in moderate-to-severe SLE [96].

### Proteasome inhibitors

Proteasomes are protein complexes that degrade unnecessary or damaged proteins by proteolysis. They are crucial for the survival and function of plasma cells, which anti-CD20 antibodies cannot eliminate due to the lack of surface CD20 [97]. Bortezomib is an N-protected dipeptide that binds to the catalytic site of the 26S proteasome. It is approved for the treatment of multiple myeloma. In Japan, studies are ongoing to investigate bortezomib in a multicenter, double-blind, randomized controlled trial in patients with refractory SLE [98].

## Conclusions and future perspectives

The data presented in this narrative review derived from original studies (31 randomized controlled trials, 25 observational and 9 basic studies), reviews of clinical or basic studies (19), systematic reviews of clinical studies (7), recommendations in SLE treatment (8) and 1 case report. Although referenced publications demonstrate diverse levels of clinical evidence, they represent very well the multidimensional picture of current and new therapeutic possibilities in SLE treatment. The clinical data reviewed indicate that establishing a standardized treatment for all patients with SLE is a challenge due to the complex pathogenesis and heterogeneity of the clinical picture of this disease. Immunological profiling and precision medicine based on transcriptome analysis can help identify immune phenotypes and gene signatures in patients with SLE [99]. This can lead to a better understanding of the pathogenesis of this disease and, consequently, improve treatment tailoring and clinical outcomes. In the long term, this modern approach may aid in selecting new treatment targets and prognostic SLE biomarkers.

## Data Availability

No data was used during preparing our article.
